# Arrhythmogenicity of the Coronary Sinus

**Published:** 2004-10-01

**Authors:** Demosthenes G Katritsis

**Affiliations:** Department of Cardiology, Athens Euroclinic, Athens, Greece

## Introduction

The coronary sinus (CS) is the cardiac venous system that begins at its ostium in the right atrium and ends at the origin of the great cardiac vein [1-3]. The major tributaries of the CS include the great cardiac vein (anterior cardiac vein), the left obtuse marginal vein, the posterior (or inferior) left ventricular vein, the middle cardiac vein, and the right coronary vein. In addition, atrial veins and, notably, the vein of Marshall (or oblique left atrial vein) also enter the coronary sinus vein [1-3].

 From the perspective of electrophysiologists, the CS represents an anatomical structure of particular interest. First, it provides access to epicardial atrioventricular pathways vein [4,5]. and arrhythmogenic foci of both atrial6 and ventricular arrhythmia vein [7,8]. Second, it represents by itself a potential source of atrial arrhythmia vein [9-14].

The arrhythmogenic potential of the thoracic veins in general has been recognised since the 1970s vein [15-16]. Atrial arrhythmias can originate in the pulmonary veins vein [17], the superior vena cava vein [18], and the CS. Indeed, biatrial flutter vein [19,20], left atrial tachycardia vein [9,10], and atrial fibrillation vein [12-14] involving the distal CS have been well described. There is now evidence that the CS apart from participating in arrhythmia circuits, such as in the slow-slow form of atrioventricular nodal reentrant tachycardia vein [21] and atrioventricular reentrant tachycardia due to accessory pathways vein [22-26], may itself be a source of apparently atrial arrhythmia. In patients with paroxysmal atrial fibrillation apparently originating from the left superior or inferior pulmonary vein, detailed epicardial mapping through the distal coronary sinus might identify epicardial location of the arrhythmogenic focus vein [6]. Therefore, the search for foci of abnormal automaticity within the CS should be part of the electrophysiologic evaluation of left atrial arrhythmias vein [13].

## The Ligament of Marshall

A potential source of arrhythmia at the CS is the area of the ligament of Marshall. The ligament of Marshall is a left atrial epicardial neuromuscular bundle that has been associated with the genesis of atrial tachyarrhythmias and AF [27,28].

**Figure 1:**
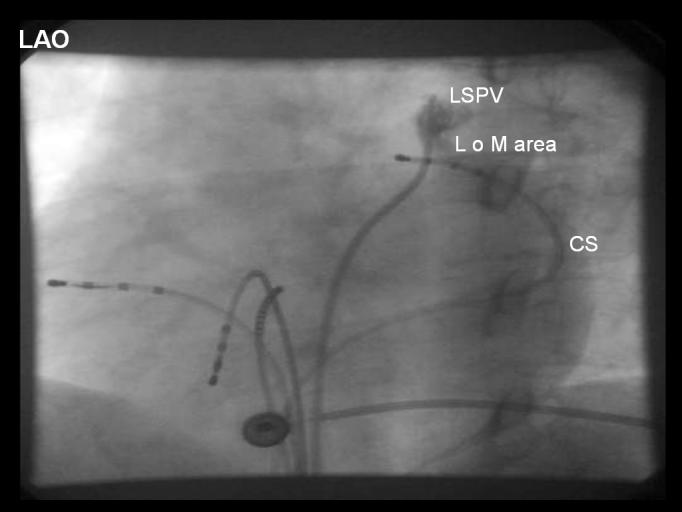
Positioning of the mapping electrode at the distal (superoposterior) CS at the epicardial aspect of the left superior pulmonary vein. Left anterior oblique 300 projection. Reproduced with kind permission from Katritsis et al. J Cardiovasc Electrophysiol 2002; 13:859-862; Blackwell Publishing.

In 1850, at The Royal Society in London, J. Marshall presented a description of a "vestigial fold of the pericardium" in the back of the auricle, running from the region of the left superior pulmonary vein to the coronary sinus [2]. During embryonic cardiac development the tributary veins of the left sinus horn are successively obliterated so that, at the 10th week, the distal part of the left sinus horn remains as the oblique vein of Marshall and the remaining proximal horn becomes the coronary sinus [2,29]. Occasionally, a left superior caval (cardinal) vein persists and drains to the coronary sinus. Normally, there is only a remnant of the left superior vena cava that persists as a fibrous cord coursing along the left side of the parietal pericardium, overlying the left pulmonary artery and joining the heart at the roof of the left atrium. This fibrous structure is readily recognised during cardiac operations and is known as the ligament of Marshall [30]. The ligament courses in the AV groove at the base of the left atrial appendage and leads to the earliest tributaries of the coronary sinus. This transition from a ligamentous structure to a vein occurs in the region between the left superior pulmonary vein and the base of the left atrial appendage. This is the corresponding atrial endocardial location for the tip of an electrophysiology catheter advanced to the "wedge" position as far as distal as possible in the coronary sinus, and, consequently, to the origin of the ligament of Marshall (Figure 1).

The ability of this structure to produce automatic activity during sympathetic stimulation was described more than a century later than its initial description. Doshi et al [28], in an animal study, clearly demonstrated that the ligament of Marshall is rich in sympathetic innervation and isoproterenol infusion may induce automatic activity from it; this sensitivity to isoproterenol is upregulated following long-term rapid pacing and may contribute to development of AF. Thus, this area may not only act as a trigger of AF paroxysms but may also result in sufficient electrical remodelling of the atria and persistent AF.

**Figure 2:**
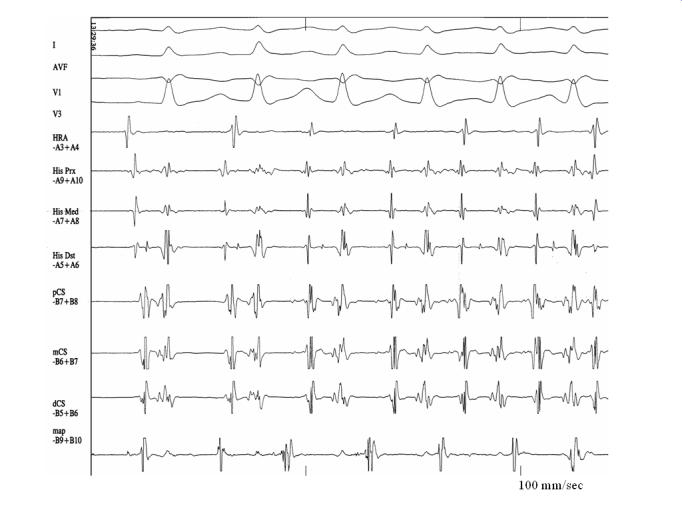
Following a sinus beat, spontaneous atrial ectopy from the ligament of Marshall area induces atrial fibrillation. HRA: high right atrium, His: His bundle, CS: coronary sinus, map: trans-septally introduced catheter just below the os of the left superior pulmonary vein and oriented towards the coronary sinus catheter (endocardial approach of the area of the ligament of Marshall). Reproduced with kind permission from Katritsis et al. J Cardiovasc Electrophysiol 2001; 12:750-758; Blackwell Publishing

We and others [12] [31] have demonstrated that electrical activity of ligament of Marshall tissue can be identified in the human by epicardial or endocardial recording of discrete potentials, and that these potentials can be selectively abolished by catheter ablation (Figure 2). Our group has also shown that combined epicardial and endocardial ablation through the coronary sinus and the left atrial endocardium is necessary in order to abolish the electrical activity of the presumed extracardiac and intracardiac components of this tissue. Abolition of this activity may significantly reduce the frequency of paroxysms of adrenergic AF and confer considerable symptomatic improvement. Kim et al [32], based on anatomical observations on postmortem human hearts, have recently suggested that the complex pattern of ligament of Marshall myocardial tract insertions into the left atrial free wall may necessitate an endocardial ablation approach with energy delivery to the lower part of the left atrium. Our observations to-date indicate that although such an approach should be part of the ablation procedure, it may not be enough without ablation of the extracardiac component. This is in keeping with observations from the surgical Maze where total isolation of the pulmonary veins has not been found enough to prevent AF unless coronary sinus cryoablation is accomplished [33].

According to our experience, cannulation of the area of the ligament of Marshall is not always possible with conventional ablation catheters. Our method requires full engagement of the distal coronary sinus with the ablation catheter and this is usually possible in approximately half of the cases. Furethermore, delivery of radiofrequency current at this area is not always feasible due to impedance rises in case of a wedged electrode. Thus, the epicardial approach may not be applicable to a considerable proportion of patients.

## The Role of Coronary Sinus Musculature

Arrhythmias originating within the CS or cardiac veins have also been attributed to atrial musculature extending into these structures. Striated myocardial connections between the venous wall of the CS and the left atrium have been described both in animal and human necropsy studies [34,35]. The myocardial sleeve around the coronary sinus is composed of bands of muscle from the left atrial wall as well as from the right atrial wall [36]. The sleeve usually does not extend to other veins, although occasionally it may cover the adjacent 2 to 10 mm of the great cardiac vein [37].

**Figure 3:**
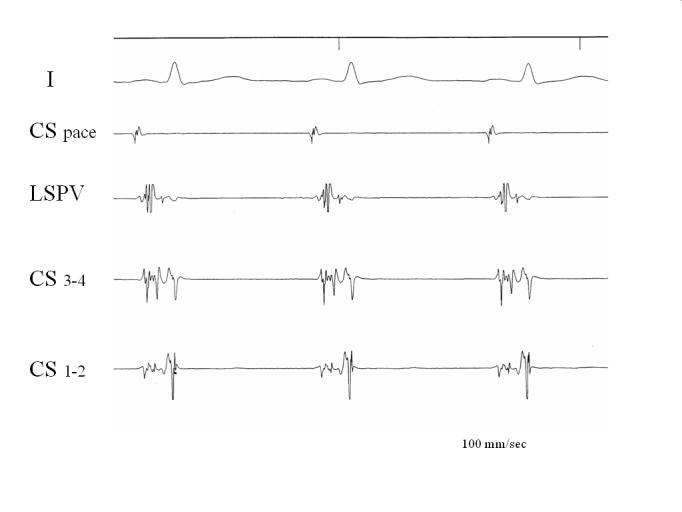
Double potentials are clearly recorded in the CS and the left superior pulmonary vein during CS os pacing. Please note clearly separated signals in dCS (CS 1-2) and fractionated activity in pCS (CS 3-4). RA: pacing catheter at right atrium, His: His bundle electrogram, CS: coronary sinus, LSPV: mapping electrode at the ostium of the left superior pulmonary vein. Reproduced with kind permission from Katritsis et al. J Cardiovasc Electrophysiol 2002; 13:859-862; Blackwell Publishing.

Myocardial connections between left atrium, pulmonary vein and CS musculature can be identified epicardially, through the CS or following a pericardiocentesis, by recording double potentials or fractionated electrograms indicative of delayed conduction [11,38-40]. We have recently shown that recording of double potentials is possible within the CS, particularly at its distal, superoposterior part, near the left superior pulmonary vein (Figure 3). Their prevalence is higher in patients with PAF than in subjects with other or no arrhythmias and their presence denotes possible sources or substrate for atrial arrhythmia [11].

The demonstration of double potentials with pacing-dependent interpotential delay, clearly suggests the possibility of conduction delay at the distal CS-LA connection. This supports the reported association of this area with the initiation of paroxysms of AF as well as macroreentrant arrhythmias. Delayed conduction at this particular site might provide the substrate for local micro-reentry or serve as a component of a macro-reentrant circuit of biatrial flutter or atrial tachycardia [9,10,19]. Our results also showed that double or fractionated potentials within the CS and in particular at the distal area, may be recorded in the human regardless of a clinical history of PAF. Thus, these potentials are not totally specific for PAF; their prevalence, however, is significantly higher in patients with PAF than in patients with other or no arrhythmias [11].

**Figure 4:**
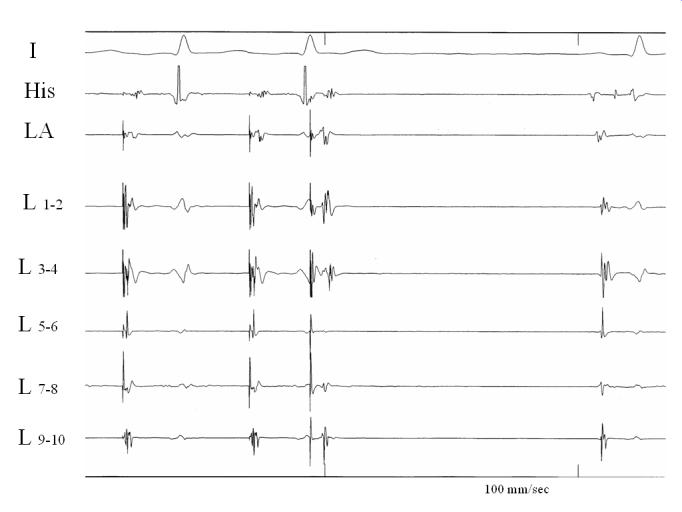
Circumferential muscle activation pattern within the coronary sinus during coronary sinus pacing. There are fused signals in bipoles 1-2, 3-4, and 9-10. Following the last paced beat (220 msec), there is atrial capture as indicated by the atrial electrogram in the His and left atrial electrodes, but the sharp potentials that were obvious in bipoles 5-6, and 9-10 have now disappeared, thus probably representing CS musculature potentials. I: lead I of surface ECG, GCV: great cardiac vein, LA: left atrium, at the left superior pulmonary vein ostium, His: His bundle electrogram, L: Lasso catheter. Reproduced with kind permission from Katritsis et al. J Interv Cardiac Electrophysiol 2004; 10: 1-8; Kluwer Academic Publishers.

If the multicomponent electrogram pattern as well as the potential arrhythmogenicity of the CS are due to myocardial connections, recording of double or fractionated potentials should not be possible in venous structures such as great cardiac vein and middle cardiac vein that lack a myocardial sleeve. We studied this hypothesis in our laborotary by subjecting 20 patients to catheter mapping of the CS, the middle cardiac vein, and the great cardiac vein. At conventional mapping during sinus rhythm and high right atrial pacing, discrete double potentials or fractionated electrograms were recorded during left, right atrial and CS pacing at the CS ostium, mid-CS, and distal CS-ligament of Marshall area, in 2 (10%), 1 (5%), and 9 (45%) patients, respectively, whereas no patient displayed such signals in the cardiac veins (p< 0.001). The pattern of circumferential muscle activation within the proximal CS was also studied with a circular mapping catheter (Lasso 12 mm). Proximal CS mapping with the Lasso was accomplished in 10 patients, 7 of whom had no evidence of multicomponent potentials in the CS at conventional mapping. Specific CS potentials dissociated from the atrial electrograms were recorded in all patiens with the use of circumferential mapping. The perimetric distribution of electrograms within the CS suggested an oblique course of conduction across the CS musculature (Figure 4).

It seems therefore that employment of more sensitive mapping techniques such as perimetric mapping, can disclose the universal existence of muscular activation around the CS in all patients tested. In addition, the pattern of activation is similar to this described by Haissaguerre et al [17] in the pulmonary vein and by Goya et al [41] in the SVC, probably reflecting the peculiar anatomy of myocardial extensions surrounding venous structures in the heart. According to von Ludinghausen’s [1,37] descriptions of the CS muscle coat as viewed from the epicardial side of the heart, the CS musculature courses across the AV groove in an oblique way. Thus an oblique rather than circular pattern of conduction should be responsible for the observed distribution of electrograms. We can speculate, therefore, that what we recorded is a combination of longitudinal conduction along the CS axis through the adjacent atrial endocardium with oblique conduction of CS musculature across the CS.

Extensive mapping of the distal, superoposterior part of the CS is technically difficult and at times impossible. Interestingly, despite conventional beliefs, in adults, venous luminal diameter is not a cause of obstruction to the passage of 6 or 7F catheters in the distal coronary sinus [43]. Our results, in keeping with post-mortem observations [1], support this view. It has been shown, both in cadaveric hearts and in clinical studies, that in the majority of the cases a first obstacle is the presence of the valve of Vieussens, and, once this has been negotiated, half of the attempts fail due to acute bending of the great cardiac vein. We have been systematically trying to map the great cardiac vein in all our electrophysiology cases the last 3 years and, despite the fact that the tributaries of the coronary sinus and of the anterior cardiac veins are very variable [42,43], our success rate now approaches 40%.

In conclusion, the discussed observations provide the rationale for the reported arrhythmogenicity of the CS itself. They support the view that atrial myocardial extensions into cardiac venous structures provide the substrate for potential arrhythmogenicity. In addition, they suggest that detailed mapping of venous structures such as the CS should be considered when assessing patients with tachyarrhythmias of apparently left atrial origin.
